# Utility of UAS-LIDAR for estimating forest structural attributes of the Miombo woodlands in Zambia

**DOI:** 10.1371/journal.pone.0315664

**Published:** 2025-03-11

**Authors:** Hastings Shamaoma, Paxie W. Chirwa, Jules C. Zekeng, Abel Ramoelo, Andrew T. Hudak, F. Handavu, Stephen Syampungani

**Affiliations:** 1 Department of Urban and Regional Planning, Copperbelt University, Kitwe, Zambia; 2 Forest Science Postgraduate Programme, Department of Plant and Soil Sciences, University of Pretoria, Pretoria, South Africa; 3 Department of Forest Engineering, Advanced Teachers Training School for Technical Education, University of Douala, Douala, Cameroon; 4 Oliver R Tambo Africa Research Chair Initiative (ORTARChI), Chair of Environment and Development, Department of Environmental and Plant Sciences, Copperbelt University, Kitwe, Zambia; 5 Centre for Environmental Studies (CFES), Department of Geography, Geoinformatics and Meteorology after CFES, University of Pretoria, Pretoria, South Africa; 6 Earth Observation Programme, South African National Space Agency (SANSA), Pretoria, South Africa; 7 USDA Forest Service, Rocky Mountain Research Station, Forestry Sciences Laboratory, Moscow, Idaho, United States of America; 8 Department of Geography, Environment and Climate Change, Mukuba University, Kitwe, Zambia; Universidade Federal de Uberlandia, BRAZIL

## Abstract

The ability to collect precise three-dimensional (3D) forest structural information at a fraction of the cost of airborne light detection and ranging (lidar) makes uncrewed aerial systems-lidar (UAS-lidar) a remote sensing tool with high potential for estimating forest structural attributes for enhanced forest management. The estimation of forest structural data in area-based forest inventories relies on the relationship between field-based estimates of forest structural attributes (FSA) and lidar-derived metrics at plot level, which can be modeled using either parametric or non-parametric regression techniques. In this study, the performance of UAS-lidar metrics was assessed and applied to estimate four FSA (above ground biomass (AGB), basal area (BA), diameter at breast height (DBH), and volume (Vol)) using multiple linear regression (MLR), a parametric technique, at two wet Miombo woodland sites in the Copperbelt province of Zambia. FSA were estimated using site-specific MLR models at the Mwekera and Miengwe sites and compared with FSA estimates from generic MLR models that employed combined data from the two sites. The results revealed that the model fit of site-specific MLR models was marginally better (Adj-R^2^: AGB =  0.87–0.93; BA =  0.88–0.89; DBH =  0.86–0.96; and Vol =  0.87–0.98 than when using a generic combined data model (AGB =  0.80; BA =  0.81; DBH =  0.85; and Vol =  0.85). However, the rRMSE (2.01 – 20.89%) and rBias (0.01-1.03%) of site specific MLR models and combined data model rRMSE (3.40-16.71%) and rBias (0.55-1.16%) were within the same range, suggesting agreement between the site specific and combined data models. Furthermore, we assessed the applicability of a site-specific model to a different site without using local training data. The results obtained were inferior to both site-specific and combined data models (rRMSE: AGB =  36.29%–37.25%; BA =  52.98–54.52%; DBH =  55.57%–64.59%; and Vol =  26.10%–30.17%). The results obtained from this indicate potential for application in estimating FSA using UAS-lidar data in the Miombo woodlands and are a stepping stone towards sustainable local forest management and attaining international carbon reporting requirements. Further research into the performance of UAS-lidar data in the estimation of FSA under different Miombo vegetation characteristics, such as different age groups, hilly terrain, and dry Miombo, is recommended.

## Introduction

Miombo ecoregion covers 10% of Africa’s land area (c: 2.5–4 million km2) [1,2], a large component of the savanna biome, contributing to the terrestrial carbon cycle and providing socioeconomic, ecological, and environmental services like climate regulation, carbon sequestration, and biodiversity conservation [3,4]. Recent climate change, attributed to increased greenhouse gas emissions, would certainly intensify climatic feedbacks such as droughts and extreme weather [5–7]. The impact of these effects on Miombo woodlands development, biomass production and carbon storage need to be adequately understood [8]. In recent decades, global climate change has highlighted the need for efficient methods to quantify and monitor forest biomass and carbon stocks at local, national, continental, and global scales [9,10].

The Reduced Emissions from Deforestation and Forest Degradation (REDD+) program promotes forest carbon conservation and enhancement in developing nations [9,11], with a focus on forest-based solutions. Most African vegetation formations lack precise forest biomass and carbon storage data needed for implementing REDD + programs. Construction of accurate models that account for current climatic conditions and diverse forest uses is necessary to predict the interactive effects of environmental changes, species distribution changes, and forest carbon storage capacity [5,12]. Forest managers need accurate and reliable estimates of forest structural attributes (FSA) in order to make educated decisions about the sustainable use of forests [13–15].

Traditional field-based sample surveys, for instance, national forest inventories, are employed to assess FSA on a regional and national level [16,17]. However, field inventory is lengthy and laborious [18–20]. Remote sensing can provide multifaceted, seamless, geographically precise observations in a quick and adaptable way for accurate FSA estimation [15,16,21–23]. Light detection and ranging (lidar) is considered the most appropriate remote sensing technology for FSA estimation, as it has the ability to capture both the vertical and horizontal traits of vegetation [24–27]. Examples of application of lidar for estimating FSA include: diameter at breast height (DBH) [27], canopy cover [28], stem density [29], basal area (BA) [29,30], volume (Vol) [31], and aboveground biomass (AGB) [32]. However, the use of lidar for forest inventory in low-income countries, where the bulk of forests are located, is limited by high data collection costs and mission safety concerns associated with piloted aircraft, which are often used [33].

The advent of Uncrewed Aerial Systems (UAS) and the advancement and downsizing of lidar sensors have made it possible to use UAS-mounted lidar systems (UAS-lidar) to assess FSA at a reduced price and with increased adaptability [33–35]. In a groundbreaking study, Lin et al. [34] developed a UAS-lidar and evaluated it for determining ground and tree heights in Vanttila, Espoo, Finland. A similar study by Wallace et al. [35] utilized UAS-lidar to determine the position, height, and crown width of trees situated at the University of Tasmania farm in Australia. Another study by Guo et al. [33] used a UAS-lidar system for estimating canopy height, canopy cover, leaf area index, and AGB in three distinct Chinese ecosystems, including a needleleaf-broadleaf mixed forest, an evergreen broadleaf forest, and a mangrove forest. Liu et al. [22] used UAS-lidar generated point clouds to estimate six FSA: DBH (r^2^ =  0.89), Lorey’s mean height (r^2^ = 0.97), stem density (r^2^ =  0.77), BA r^2^ =  0.89), Vol (r^2^ =  0.94) and AGB (r^2^ =  0.95) in Pizhou Ginkgo plantations, China. Studies by Wallace et al. [22], Guo et al. [33] and Liu et al. [35] have demonstrated the capability of UAS-lidar point clouds for estimating various forest attributes that are critical for forest management. However, none of the studies were carried out in African savannas or Miombo woodlands in particular.

Thus far only a few studies have employed remote sensing methods in estimating AGB in the Miombo ecoregion. For example, Kashindye et al. [36] employed medium-resolution Landsat imagery and MLR to estimate FSA in the Miombo woodlands of Bereku and Duru Haitemba forests in Tanzania. Another study by Halperin et al. [37] used Landsat imagery, a semiparametric generalized additive model (GAM), and two nonlinear models (sigmoidal and exponential) to predict AGB in the Miombo woodlands of Nyimba District in Zambia. Recently, Macave et al. [23] used a combination of optical (Landsat 8/OLI and Sentinel 2A/MSI) and radar (Sentinel 1B and ALOS/PALSAR-2) data to estimate AGB in the Miombo Woodlands in Niassa Special Reserve, Northern Mozambique. However, the AGB estimates from imagery ulitised in studies by Kashindye et al. [36], Halperin et al. [37] and Macave et al. [23] fall short of the precision required for international reporting mechanisms and sustainable forest management at a local level [38,39]. Airborne lidar data, which overcome the shortcomings in imagery that were utilized by Kashindye et al. [36], Halperin et al. [37] and Macave et al. [23] were used by Mauya et al. [40] to estimate AGB in the Miombo woodlands of Tanzania with sufficient precision (rRMSE =  46.8%) for international reporting mechanisms and sustainable local forest management. They compared the parametric linear mixed effects (LMM) and non-parametric k-nearest neighbor (k-NN) models and revealed that both approaches are applicable for predicting AGB in the Miombo woodlands. However, the cost of acquiring airborne lidar is prohibitive for most forest managers in the Miombo ecoregion [41], and this study only focused on the estimation of AGB, leaving out the other FSA that are also important for forest management [13,14].

A study by Kachamba et al. [42] utilized cheaper UAS imagery and Structure from Motion (SfM) (UAS-SfM) derived point clouds to estimate AGB using MLR models in the Miombo woodlands of Muyobe forest, Mzimba District, in northern Malawi, to levels of precision (rRMSE = 46.7%) sufficient for international reporting mechanisms and sustainable local forest management. Nevertheless, the UAS-SfM approach has been reported to perform poorly in denser forest environments [43], which would make it challenging to promulgate to denser parts of the Miombo woodlands. Furthermore, studies by Mauya et al. [40] and Kachamba et al. [42] were based on models developed from data collected from a single site; the estimation of FSA using models developed using data from separate sites as demonstrated in earlier studies [29,30,44,45] in other vegetation formations, is yet to be investigated in the Miombo woodlands.

Therefore, this study explored the use of UAS-lidar for supplementing and filling the gaps in other remote sensing imagery that have been used in predicting FSA (AGB, BA, and Vol) in Miombo woodlands. UAS-lidar from two sites, 95 km apart, and area-based methods were used to estimate FSA using multiple linear regression (MLR) models in the wet Miombo woodlands in the Copperbelt Province of Zambia. This modeling technique was chosen because it has been shown to perform well even on small sample data sets [46,47], as was the case in this study. Furthermore, our choice was motivated by the recommendation by Næsset et al. [48] that MLR is the method of choice for realistic forest inventories. To accomplish this, we attempted to answer the following questions:

i How can UAS-lidar be used to improve FSA estimations in the Miombo ecoregion region?
ii How does the performance of the regression model created from a combination of data from two sites, located 95 km apart, compare to that of the model created from a single site?
iii Can models developed at a particular site be extrapolated to another site in the absence of ground reference data?

## Materials and methods

### Ethics statement

The fieldwork was carried out in designated national forests under the management of the Zambia Forest Department. Permission to conduct the described field studies, involving the collection of field a samples was granted by the Zambia Forest Department. The sampling did not involve disturbing endangered or protected plant or animal species in the study area. The field crew members depicted in Fig. 3 provided their consent for their pictures to appear in the paper.

**Fig 1:**
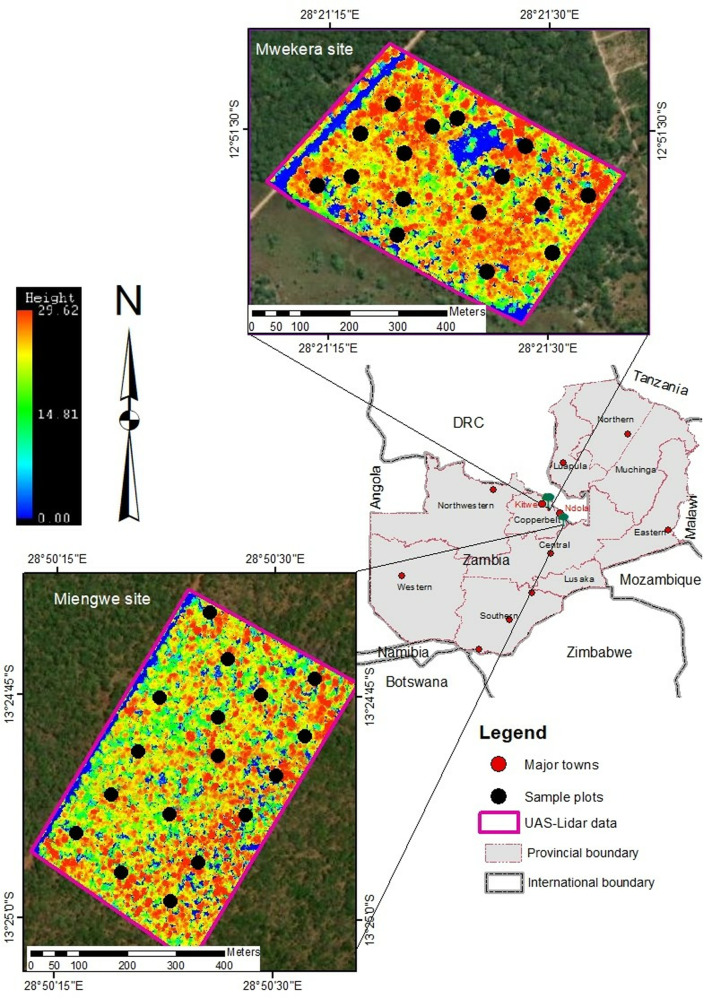
Location of study sites and distribution of sample plots (Map of Zambia shape files with permission from Republic of Zambia Survey Department).

### Study area

The study was conducted at two test sites (Fig 1). The first one was undertaken in Mwekera national forest reserve number 6 (12.860977^o^ S, 28.357049^o^ E at mean altitude of 1225 m above mean sea level), in Kitwe district, about 15 km southeast of the central business district (CBD). Mwekera forest encompasses approximately 11,100 hectares. The other study site was located in Miengwe forest reserve number 36 (13.413889^o^ S, 28.838889^o^ E and mean altitude of 1328 m above mean sea level), in the Masaiti district, approximately 90 km southwest of the city of Ndola CBD and about 17 km off the Ndola – Lusaka highway. Miengwe forest reserve covers an estimated area of 8,094 hectares. Both forests are located in the Copperbelt Province of Zambia, which experiences annual precipitation between 1000 and 1500 mm and temperatures between 25 and 32 degrees Celsius. The Miombo woodlands, which cover roughly 45 percent of Zambia’s area, are the predominant vegetation in these forests not only in Zambia but across southern Africa [49,50]. The woodlands are characterised by the dominance of three key deciduous genera (*Brachystegia, Julbernadia* and *Isoberlinia*) belonging to the family Fabaceae, subfamily Caesalpinioideae [51], though the dominant species vary (See Tables 1 and 2).

**Table 1:** Summary of diameter at breast height (DBH) and tree height (TH) data collected for 10 most dominant species at Mwekera site.

Tree Species	N	%	DBH (cm)		TH (m)	
		Abundance	Mean	Range	Mean	Range
*Julbernardia paniculata*	127	18.5	31.03	13.5 - 59.90	17.79	8.50 - 25.00
*Isoberlinia angolensis*	114	16.6	23.92	9.90 - 44.70	14.55	5.00 - 20.50
*Marquesia macroura*	108	15.7	29.21	5.30 - 70.00	15.10	3.25 - 25.00
*Brachystegia longifolia*	64	9.3	20.65	11.8 - 64.00	11.27	8.50 - 23.00
*Brachystegia spiciformis*	51	7.4	18.55	5.00 - 64.20	9.97	5.80 - 20.50
*Parinari curatellifolia*	18	2.6	23.48	6.00 - 53.50	13.67	6.00 - 24.00
*Ochna pulchra*	17	2.5	7.62	5.20 - 10.90	5.70	4.50 - 8.00
*Baphia bequaertii*	16	2.3	11.63	5.80 - 23.70	6.95	3.00 - 15.00
*Pericopsis angolensis*	16	2.3	24.42	10.3 - 70.00	14.01	5.00 - 25.10
*Diplorhynchus condylocarpon*	14	2.0	8.94	5.00 - 18.00	7.64	4.50 - 10.00

**Table 2:** Summary of diameter at breast height (DBH) and tree height (TH) data collected for 10 most dominant species at Miengwe site.

Tree Species	N	%	DBH (cm)		TH (m)	
		Abundance	Mean	Range	Mean	Range
*Brachystegia longifolia*	112	22.1	22.6	7.7 - 81.0	13.68	5.0 - 27.1
*Diplorynchus condylocarpon*	68	11.4	9.4	5.0 - 30.9	8.26	5.6 – 15.0
*Baphia bequaertii*	56	9.3	12.8	5.2 - 25.1	7.78	5.7 – 11.0
*Isoberlinia angolensis*	48	8.1	17.9	6.4 - 59.2	25.50	7.1 – 22.0
*Pseudolachnostylis maprouneifolia*	36	6.0	13.6	5.2 - 24.7	8.33	5.2 - 11.2
*Combretum zeyheri*	32	5.3	9.2	5.1 - 15.3	9.05	6.2 – 12.0
*Julbernadia paniculata*	29	4.9	41.7	20.0 - 98.3	16.88	13.0 - 25.2
*Pericopsis angolensis*	23	3.9	20.8	6.4 - 47.0	11.33	5.1 - 19.8
*Ochna schweinfurthiana*	22	3.7	8.8	5.8 - 13.5	7.61	5.8 - 12.0
*Combretum collinum*	22	3.7	11.4	5.5 - 21.7	9.73	6.0 - 14.2

**Fig 2:**
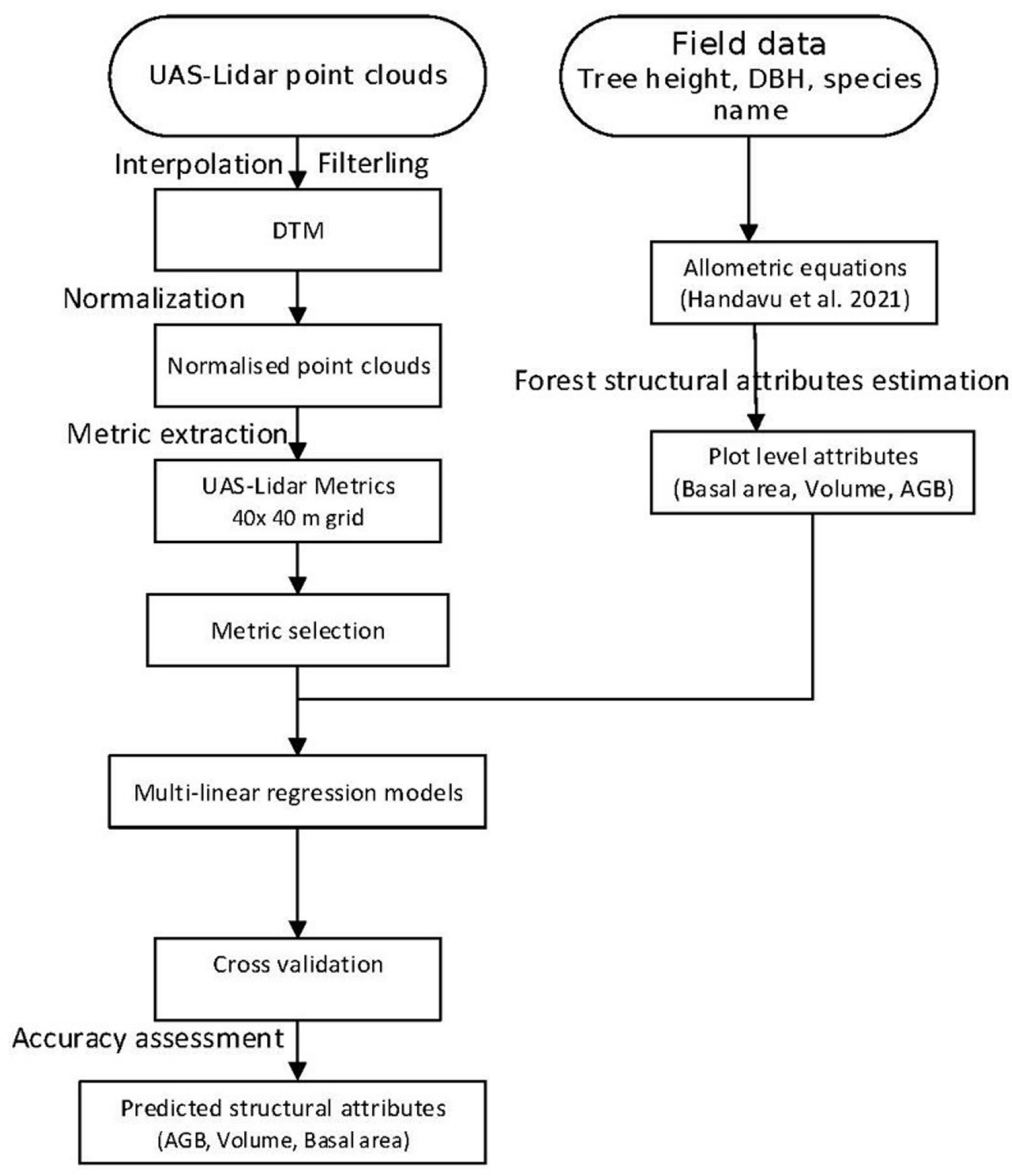
Forest structural estimation workflow.

**Fig 3:**
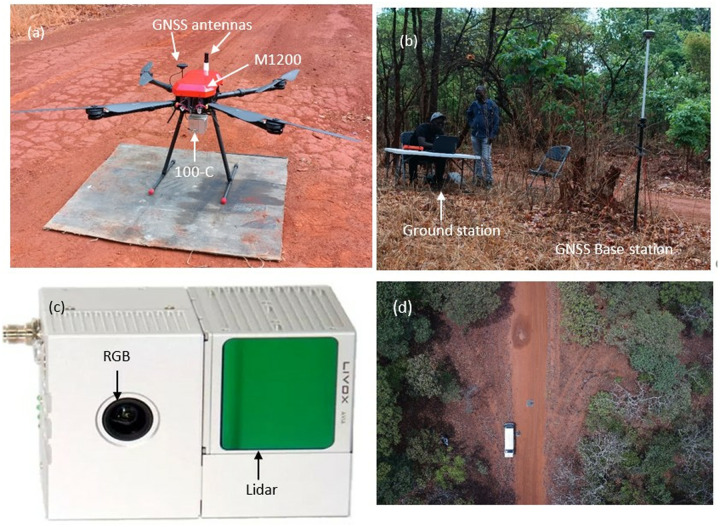
(a) T-Drone M1200 platform with 100-C sensor, (b) ground station and GNSS base station, (c) 100-sensor, and (d) aerial view of launch station.

### Data collection

An overview of the data collection and processing workflow is shown in Fig 2 and detailed in the following sections

### Field data collection

Field work was conducted in November 2022 with sixteen [16] circular plots (radius =  20 m) established at 200 metre spacing and in areas characterized by abrupt fluctuations in vegetation coverage. The coordinates of each plot centre were located using a LT700H real-time kinematic (RTK) Global Navigation satellite system (GNSS) handheld tablet (Shanghai Heave Navigation Technology, China) receiving real-time differential signals from a Continuously Operating Reference Station. All trees in the marked plots with DBH greater than 5 cm were identified to the species level, and their DBH and total height (TH) were measured. The DBH was measured using a diameter tape and TH was measured using a Nikon Forest Pro hypsometer. The measurements of the ten most dominant species for the two sites are summarized in Table 1 and 2.


$$\ln\left(AGB\right)=\ln\left(a\right)+bln(\rho D^{2}H)+\varepsilon$$ (1)

In this study, the mean BA, Vol, and AGB were determined by aggregating individual tree data at the plot level. Estimates of each of the FSA for the training data was calculated using the following variables: DBH (basal area), DBH & TH (volume) and DBH, TH & wood density (AGB). The wood density (g/cm3) values were generated using species and genus wood density values from two data sources namely [50], and the ICRAF database (www.worldagroforestry.org/wd/genus). For species that were not directly recorded in the above data sets, average wood density of the genus species was calculated. Standing tree volumes were calculated using three data parameters namely DBH, TH and tree form factor (0.74). Estimations of AGB were based on the best-fit models developed by Handavu et al. [50] within the same site (equation 1).

where AGB = total AGB (kg/tree), D = DBH, and H = tree height. The variables a, b, c, and d are model parameters. “ln” stands for the natural logarithm, while “e” represents a random error.

### UAS Data collection

A T-Drone M1200 quadcopter with a gAirHawk GS-100C UAS-lidar scanning system (comprising an integrated Livox new generation laser scanner, GNSS and IMU positioning and attitude determination system, and a storage control unit) were used. The Livox Avia sensor on the GS-100C UAS-lidar operates at 200 HZ and can provide up to 720,000 points/sec in triple echo. Mission Planner open-source software was used for flight planning and to continuously track the aircraft and monitor flight parameters of the system. In addition, a GNSS ground reference station was used to provide accurate reference measurements and other parameters for post processing the UAS-lidar data (Fig 3). The hardware setup for the system is shown in Fig 3, while the specifications for T-Drone M1200 and GS-100C are shown in Table 3. The GS-100C lidar system is capable of recording up to three returns per pulse at a near-infrared wavelength of 905 nm (Table 3).

**Table 3:** T-Drone M1200 and GS-100C sensor specifications.

T-Drone M1200 Specifications		
Maximum (kg)	5	
Maximum flying weight (kg)	18.5	
Maximum flying time (min)	60	
Flying distance (km)	10	
Flying height (m)	1000	
Flying speed (ms^-1^)	10	
GS-100C Specifications		
Weight (Kg)	1.1	
Lidar unit	Lidar class	905 nm Class 1
	Range accuracy	1σ (@20 m) < 2 cm
	Data	Triple echo, 720000 points/sec
	FOV	70⁰ the circular view
	Laser sensor	Livox Avia
POS Unit	Update frequency	200HZ
	Pitch accuracy	0.025⁰
	Roll accuracy	0.025⁰
	Heading accuracy	0.080⁰
	Positional accuracy	0.02 ~ 0.05 m
Camera	Camera model	Sony a 6000 (Non standard)
	Effective pixel	24 Mega pixel
	Trigger event	Distance or time

### Flight planning and data acquisition

The raw UAS-lidar point clouds were acquired on 9^th^ November 2021, after leaf-flush of the dominant Miombo trees and before emergence of the herbaceous layer. This date was chosen to enable the capture of a well-defined canopy and minimum herbaceous coverage for accurate normalized point cloud derivation, which is essential for the subsequent generation of lidar metrics and FSA modeling. The lidar data were acquired at a flight altitude of 80 m above ground level, flight speed of 5 m·s^ − 1^, and swath width of 42 m.

### Lidar data pre-processing

The first pre-processing stages of the collected UAS-lidar data were done in gAirhawk 5.0 version software (Geosun Navigation Technology Limited, Wuhan, China), where lidar data, IMU data, and GNSS base data were integrated to process the flight trajectory and generate georeferenced UAS-lidar point cloud data in las format. Additionally, the UAS-lidar point clouds were denoised using an outlier removal algorithm in Lidar360 software (GreenValley International, California, CA, USA). The algorithm utilizes adjacent data points and a multiple of the standard deviation to detect anomalous data points. The study utilized the enhanced progressive Triangulated Irregular Network (TIN) densification (IPTD) filter algorithm to differentiate between ground points and non-ground points [52]. Then the inverse distance weighting (IDW) interpolation algorithm was used to generate the Digital Terrain Model (DTM), which was subtracted from each point’s elevation value to produce normalized point clouds [53].

### Extraction of UAS-lidar metrics

Lidar metrics are data derivatives commonly used as predictors in regressions of FSA on lidar metrics [22,28,54]. An area-based technique using a 40 x 40 m grid was used to generate several UAS-lidar metrics (Table 4), which were then regressed against matching 20 m radius field plot data to estimate plot level FSA (Fig 2). A thorough explanation of the lidar metrics may be found in [55]. To guarantee that non-canopy returns were excluded, a 2 m height threshold was used during metric extraction [56].

**Table 4:** Description of metrics derived from UAS-lidar data.

Lidar metrics	Description
Percentile heights (H1, H5, H10, H20, H25, H30, H40, H50, H60, H70, H75, H80, H90, H95, H99)	The percentile of the canopy height distributions (1^st^, 5^th^ 10^th^, 20^th^, 25^th^, 30^th^ 40^th^, 50^th^, 60^th^, 70^th^,75^th^, 80^th^ 90^th^, 95^th^ and 99^th^) of first returns
Canopy return density (D1, D2, D3, D4, D5, D6, D7, D8, D9)	The percentage of points above the quantiles (10^th^, 20^th^, 30^th^, 40^th^, 50^th^, 60^th^, 70^th^, 80^th^ and 90th) to total number of points
Variance of height (Hvar)	The variance of the heights of all points
Maximum height (Hmax)	Maximum of return heights above 2 m
Coefficient of variation of heights (Hcv)	Variation of heights of lidar returns above 2 m
MADMedian (Hmdm)	The median of median absolute deviation
Hmsq	Generalized means for the second power
Hskew	Skewness of height
Hkurtosis	The kurtosis of the heights of all points
Hstd	Standard deviation of height
Hmean	Mean height above ground of all first returns
Canopy relief ratio (CRR)	mean height returns minus the minimum height divided by the maximum height minus the minimum height
Canopy cover (CC) above 2 m	Percentile of first returns above 2 m
Gap fraction (GF)	An indication how much of the sky is visible from beneath a plant canopy.
Leaf area index (LAI)	Half of the surface area of all leaves per unit ground area

### Development of forest structural estimation models

As per the findings of previous studies [57,58], it has been observed that the utilization of distinct modeling methodologies can yield varying outcomes. This study developed MLR models for estimating Miombo woodlands FSA based on extracted UAS-lidar metrics. Three key steps were followed in the modelling processes which included: (i) variable selection, (ii) model development/ fitting, and (iii) model validation. The details for each step are described below.

### Variable selection

The area-based prediction of forest inventory properties is based on statistical relationships between the predictor variables, which are lidar metrics, and the response variables, which are plot-level FSA [59–61]. The objective is to choose a superior subset of variables with the aim of maximizing the predictive efficacy of the model [59]. The motivations for variable selection include enhancing universality, minimizing time, and simplifying comprehension [61]. The present study scrutinized the correlation between FSA and several UAS-lidar metrics using Pearson’s correlation coefficient (r), and multi-collinear variables (r >  0.85) were excluded to facilitate model parsimony and minimize overfitting. The best subsets regression strategy was used to identify the most appropriate linear models for predicting the FSA based on selected variables [30], which was implemented in Minitab Version 21.1.1 (Minitab, 2023) to select the best performing model and variables. The module explores different combinations of variables in order to create subsets for regression models. These subsets are then assessed and ranked using multiple scoring criteria, such as R^2^, Akaike’s Information Criterion corrected (AICc), Bayesian information criterion (BIC), and Mallow’s Cp statistics [62]. In the present work, the AICc criterion, which has shown superior performance for smaller sample sizes [63,64], was prioritized above other criteria in the determination of the best regression model. The selection of the optimum subset for model building included considering a combination of predictors that minimized AICc over each of the possible subsets.

### MLR model

The MLR method has been widely used in the estimation of FSA because of its ability to handle dependencies or correlations between the predictor variables [30,57,65]. MLR assumes a linear relationship between a dependent variable (e.g., AGB, BA and Vol) and a set of independent variables (lidar metrics). The natural logarithm data transformation was applied to the dependent variable to improve the model fitting in line with previous studies [17,30]. The predictions obtained were subjected to a back-transformation process by exponentiation [66]. The log transformation introduces a systematic bias, which was corrected during the exponentiation using a bias correction factor based on half the mean squared error [30] The MLR model with the lowest AICc for each FSA was implemented.

### Model performance

The model performances were evaluated based on differences in the R^2^ and RMSE.


$$RMSE=\sqrt{\overset{n}{\underset{i=1}{\sum }}\frac{{\left(y_{i}-{\hat{y}}_{i}\right)}^{2}}{n}}$$ (2)

$$rRMSE=\frac{RMSE}{\hat{y}}x100\%$$ (3)

$$Bias=\overset{n}{\underset{i=1}{\sum }}\frac{{\left({\hat{y}}_{i-}y_{i}\right)}^{}}{n}$$ (4)

$$rBias=\frac{Bias}{\hat{y}}x100\%$$ (5)

Where yi and ŷi denote field measured FSA and predicated FSA for plot i, respectively and n is the number of measured FSA. K-fold cross validation was used to compare the developed MLR and SVR models and understand their performance. This method entails randomly dividing the data into k approximately equivalent folds or groups. In k iterations, each of these folds is regarded as a validation set. We used a k-value of 10 because it has been widely used and empirically demonstrated to produce test error rate estimates with neither excessively high bias nor extremely high variance. The dataset was divided into 10 subsets for the 10-fold cross-validation. One subset was kept aside in each fold and used to evaluate the trained model (the validation set), while the remaining 9 subsets were used for training. Once a subset has been used for validation, the process is repeated until all subsets have been used. Finally, the predicted values from all the folds were compiled into a table, and the equations presented above were applied to the table to estimate cross validated RMSE.

## Results

### Variable selection

The selection of predictor variables (UAS-lidar metrics) was conducted separately for each dependent variable (AGB, BA, DBH, and Vol). The predictors that were selected comprised a combination of parameters relating to height, density, and canopy cover. Table 6 indicates that among the metrics associated with canopy, CC was the most often chosen in the majority of the models. In terms of metrics related to height, H25 and H80 were the most commonly picked. Additionally, for metrics related to density, D60 emerged as the most frequently chosen metric. The selected models and variables are highlighted in Table 5. The models with a smaller number of predictors were prioritized over models with a larger number of predictors due to their demonstrated stability and ability to mitigate overfitting [30,67]. For example, when estimating the AGB, the model with four predictor variables was chosen based on lower AICc over the first model with five predictors, despite the latter exhibiting higher R^2^ and adjusted R^2^ values, as well as lower AICc values.

**Table 5:** Candidate models for field estimated forest structural attributes prediction using UAS-lidar metrics (see table 3 for UAS-lidar metrics description).

Vars	R^2^	R^2^ adj	R^2^ pred	Cp	AICc		BIC		CC		CRR		Haad		H25		H50		H80		H99		Hcv		Hstd		Hmdm		Hm		Hmd		D10		D30		D40		D60	
Above ground biomass																																								
1	0.52	0.50	0.36	21.2		255.824		258.508								X																								
2	0.71	0.68	0.60	3.9		248.849		251.977										X																				X		
3	0.75	0.72	0.60	3.0		247.057		250.347												X		X						X												
4	0.83	0.80	0.67	2.1		243.824		245.419		X										X		X						X												
5	0.84	0.80	0.68	2.2		243.351		245.936		X										X		X						X		X										
6	0.86	0.72	0.56	10.6		244.724		246.412		X										X		X		X				X						X						
7	0.91	0.77	0.62	12.0		247.354		247.423		X				X						X				X						X		X		X						
8	0.93	0.80	0.61	12.9		251.627		249.542		X				X						X				X						X		X		X		X				
9	0.93	0.80	0.60	13.6		256.438		251.420		X				X						X				X						X		X		X		X				X
10	0.94	0.70	0.42	18.405		263.081		254.178		X				X		X		X		X		X		X				X				X		X						
Basal area																																								
1	0.50	0.48	0.38	175.26		177.77		32.4								X																								
2	0.59	0.56	0.47	173.17		176.04		24.8												X																		X		
3	0.65	0.61	0.56	172.17		175.11		20.1												X		X						X												
4	0.73	0.67	0.61	169.71		172.36		14.2		X										X		X						X												
5	0.81	0.76	0.70	164.64		166.59		7.4										X				X				X		X								X				
6	0.85	0.80	0.73	163.16		163.91		5.1		X										X		X				X		X								X				
7	0.89	0.79	0.72	164.50		164.47		3.3		X				X						X		X				X		X								X				
8	0.90	0.78	0.68	164.39		160.86		4		X		X		X						X		X				X		X								X				
9	0.91	0.80	0.71	166.62		159.72		4.3		X		X		X						X		X				X		X								X				X
10	0.93	0.88	0.79	170.07		158.70		4.8		X		X		X						X		X				X		X				X				X				X
Diameter at breast height																																								
1	0.66	0.65	0.58	143.39		145.90		21.4										X																						
2	0.78	0.76	0.70	136.14		139.02		9.9														X		X																
3	0.86	0.84	0.79	128.03		130.96		2.2		X												X		X																
4	0.88	0.85	0.80	127.84		130.49		1.8		X												X		X																X
5	0.88	0.85	0.78	131.21		133.15		3.5		X												X		X								X								X
6	0.89	0.85	0.76	134.17		134.92		4.7		X						X				X		X		X																X
7	0.89	0.84	0.72	138.71		137.68		6.4		X						X		X				X		X								X								X
8	0.92	0.87	0.55	137.60		134.08		5.2		X						X		X		X		X				X				X		X								
9	0.93	0.88	0.75	140.72		133.82		5.9		X				X		X		X		X		X				X				X		X								
10	0.928	0.877	0.615	147.69		136.31		7.6		X				X		X		X		X		X				X				X		X								X
Volume																																								
1	0.72	0.70	0.59	171.84		174.35		8.1								X																								
2	0.80	0.78	0.75	166.50		169.37		9.2								X																		X						
3	0.85	0.83	0.78	162.19		165.12		7.4		X						X																		X						
4	0.88	0.86	0.81	146.44		140.34		3.6		X						X								X										X						
5	0.91	0.78	0.74	148.01		140.64		6.9		X				X		X												X								X				
6	0.93	0.71	0.69	147.24		148.74		7.7		X				X						X		X						X								X				
7	0.95	0.73	0.67	152.60		151.57		9.4		X				X						X								X		X		X				X				
8	0.97	0.75	0.69	159.63		162.28		12		X				X				X		X								X		X		X				X				
9	0.97	0.76	0.62	156.91		158.85		15.4		X		X		X						X		X						X						X		X				
10	0.98	0.86	0.68	154.89		155.64		21.8		X		X		X				X		X		X						X						X		X				

**Table 6:** Summary of cross validation results of model for R^2^, RSME and rRSME, Bias and rBias.

Site	Response variable	Prediction equation	R^2^	R^2^_adj	RMSE	rRMSE (%)	Bias	rBias (%)
Mwekera	ln(AGB)	1.42CC + 0.02H25 + 1.27Hcv - 3.57D20 - 1.27D60 + 0.24	0.90	0.87	15.41	15.46	0.01	0.01
	ln(BA)	1.12LAI + 1.24CRR - 0.02H25 + 0.05H80 - 3.05Hcv + 9.16D10) + 1.96D60 -0.98	0.92	0.88	1.78	11.12	-0.01	-0.04
	ln(DBH)	0.25LAI – 0.05Haad + 1.12CRR + 0.01H25 + 0.08Hstd – 0.31D80 + 0.32	0.90	0.86	1.51	6.37	0.05	0.19
	ln(Vol)	1.05LAI + 2.16CRR - 2.71Hcv + 0.13Hstd + 8.98D10 + 1.94D60 - 0.21	0.94	0.87	28.29	14.08	2.06	1.03
Miengwe	ln(AGB)	2.52CC - 8.27Hcv + 0.85Hstd + 5.49D20 – 1.84D50 - 0.07	0.95	0.93	15.38	14.76	-0.73	-0.70
	ln(BA)	2.19CC – 5.71Hcv + 1.83Hstd – 1.10Haad – 3.12D50 – 1.44	0.92	0.89	2.81	20.89	1.63	0.10
	ln(DBH)	0.62CC + 0.03H1 – 0.04H25 – 2.70Hcv + 0.20Hstd – 0.012D70 + 1.237	0.97	0.96	0.31	2.01	0.00	0.01
	ln(Vol)	1.81CC – 0.79Haad – 7.33Hcv + 1.58Hstd + 3.16d20 -2.83D50 + 0.67D60 = 0.04	0.99	0.98	12.15	9.50	-0.73	-0.57
Combined	ln(AGB)	0.87CC + 0.13H80 – 0.06H99 – 0.19Hmd + 0.73	0.83	0.80	18.39	16.05	1.26	1.10
	ln(BA)	0.95CC + 0.17Haad + 0.07H80 + 0.07H99 + 0.38Hstd -6.62D30 -0.04	0.85	0.81	2.69	16.71	0.19	1.16
	ln(DBH)	0.48CC + 0.03H99 -0.67Hcv + 0.26D60 + 0.51	0.87	0.85	2.27	11.86	0.11	0.55
	ln(Vol)	1.21CC + 0.07H25 + 1.40Hcv -6.27D10 + 0.37	0.88	0.85	5.94	3.40	1.38	0.79

### MLR forest structural attribute estimations

**Fig 4:**
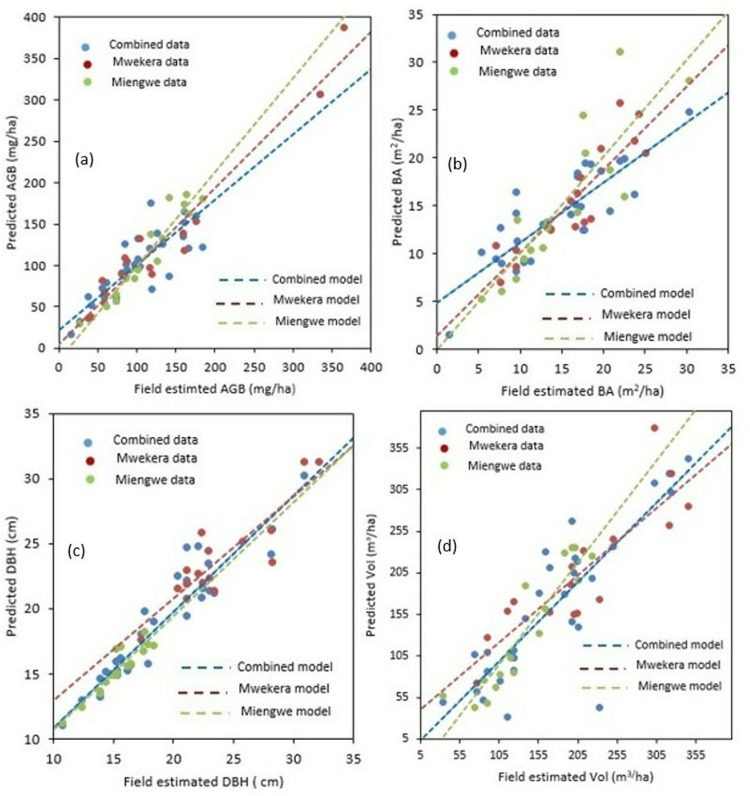
Shows a comparison of UAS-lidar estimated and field estimated FSA for Mwekera and Miengwe sites utilizing single site data models and combined data models. (a) above ground biomass, (b) basal area, (c) diameter at breast height and (d) volume.

The modeling results are shown in Table 6 and Fig 4. In general, the predictive performance of site-specific models for FSA was superior (Adj-R^2^: AGB =  0.87–0.93; BA =  0.88–0.89; DBH =  0.86–0.96; and Vol =  0.87–0.98) to that of the combined data model (AGB =  0.80; BA =  0.81; DBH =  0.85; and Vol =  0.85). In addition, this research evaluated the transferability of the site-specific models between Mwekera and Miengwe (as shown in Table 7) and found that these models exhibited lower levels of model fit (R^2^ =  0.41-0.54) compared to both the site-specific models (R^2^ =  0.94-0.99) and combined data models (R^2^ =  0.83-0.88).

**Table 7:** Local model transferability R^2^, RMSE and rRSME, Bias and rBias.

Site	Attributes	Model	R^2^	RMSE	rRMSE(%)	Bias	rBias (%)
Mwekera	AGB	Miengwe	0.49	37.82	36.29	9.45	9.07
	BA		0.41	7.30	52.98	-5.12	-37.16
	DBH		0.51	8.44	55.57	7.74	50.96
	Vol		0.50	33.39	26.10	12.76	9.97
Miengwe	AGB	Mwekera	0.53	38.82	37.25	9.70	9.31
	BA		0.45	7.51	54.52	1.63	11.86
	DBH		0.54	9.81	64.59	-3.00	-19.74
	Vol		0.43	38.17	30.17	9.65	7.54

## Discussion

### Selecting the best predictors for estimating FSA

We undertook variable selection to choose the best possible predictors (UAS-lidar metrics) to estimate the FSA of interest (AGB, BA, DBH or Vol). The best predictors for each forest structural attribute were a mix of height, density, and canopy cover-related metrics (Table 5). The height percentiles were the most selected lidar metrics across all FSA. This is consistent with earlier observations that demonstrated that height-related metrics (particularly high percentiles) are important in estimating FSA [22,68–70]. However, there is a noticeable variation in the selected predictors for the two sites (site equations in Table 6) despite both being the wet Miombo woodlands. The observed differences may be ascribed to disparities in the structure and composition of the forests at the two locations, as seen in Tables 1 and 2. This finding is consistent with a previous investigation conducted by Bouvier et al. [70].

### Site specific vs combined data models

The results presented in Table 6 demonstrate that the Mwekera and Miengwe site-specific models exhibited superior model fit (adj-R^2^ =  0.86-0.98) compared to the combined site data model (adj-R^2^ =  0.80-0.85), confirming assertions by [71]. This agrees with claims by Bouvier et al. [70] regarding the prominence of the dominant species in a forest stand, which significantly influences the correlation between lidar metrics and the FSA that is being estimated through area-based lidar techniques employed in this study. In relation to accuracy, the site-specific models (rBias: 0.01-1.03%) exhibited a similar range to the combined data model (rBias: 0.55-1.16%), indicating coherence between the site-specific and combined data models. This contradicts work by Bouvier et al. [70], who found that site specific models yielded better estimates of FSA than large-areas generic models. The congruity between the site-specific model and the integrated data model may be ascribed to the resemblance in the vegetation structure of the two sites used in this study, since they were both Wet Miombo sites (Tables 1 and 2).

### Site-specific model transferability

We assessed the efficacy of extrapolating single site models to estimate FSA in regions where UAS-lidar data is available, but field forest inventory measurements are assumed to be unavailable (Table 7). The obtained results (rRMSE =  26.10%-64.59%) fall within a similar range observed by [29]. In their study, Fekety et al. [29] evaluated the transferability of random forest regression models in estimating BA and Stem density (rRMSE =  32.3%-67.3%) across six lidar sites located in the Northern Rocky Mountains, Idaho, United States of America. In the present study, the performance of a transferred local model was found to be suboptimal when applied to a new site lacking field training data (Table 7) in comparison to the site where it was originally developed (Table 6). As such, this finding may serve as a starting step for forest managers wishing to incorporate UAS-lidar data gathered for non-forest applications into forest inventory processes. Nonetheless, the amalgamation of UAS-lidar collections from multiple sources presents challenges in terms of data quality. This is primarily due to the utilization of different flight settings and sensor characteristics [72–74]. Consequently, forest managers wishing to combine USA-lidar from various sources are advised to employ a cautious, consistent approach to calculate reliable lidar metrics [29].

### UAS-lidar improved FSA estimates

This study used UAS- to estimate more FSA (AGB, DBH, Vol and BA) compared to earlier studies that were carried out in the Miombo, which focused on AGB [40,42]. We were able to achieve superior estimations of AGB: rRMSE =  12.17% - 20.71%, compared to rRMSE =  46.8% reached by Mauya et al. [40] in Tanzania using airborne-lidar and rRMSE =  46.7% achieved by Kachamba et al. [42] in Malawi using UAS-SfM. The difference in AGB estimation accuracy from Mauya et al. [40] was most likely caused by the differences in point cloud densities. The UAS-lidar used in this study had an average point density of about 300 pts m^-2^, compared to the manned airborne lidar system used in Mauya et al. [40] with an average point density of about 1.8 pts m^-2^. An increased point density results in enhanced delineation of the canopy structure, consequently leading to more accurate estimations of AGB [75]. The discrepancy in findings from Kachamba et al. [42] may be due to the better DTM from UAS-lidar employed in this work, which has the capacity to penetrate the canopy and capture the vertical distribution of the canopy, as compared to UAS-SfM used in [42]. This conforms with findings from studies by Wallace et al. [76] and Cao et al. [66], who compared UAS-lidar and UAS-SfM for estimating FSA in a dry sclerophy11 eucalypt forest in Australia and in a planted forest in China, respectively, and found that UAS-lidar yielded better estimates, due to the superior canopy penetration capability of active lidar technology versus the passive optical stereo imagery used in the UAS-SfM technique.

Although our study was confined to just two sites, site specific studies on a small number of species are critical to updating current knowledge and information thereby aiding in the sustainable use and management of forest resources [4]. As a result, the techniques adopted here may be replicated in other parts of the Miombo with comparable vegetation formations. Further, considering the endurance and storage challenges that continue to plague UAS-lidar technology [41,77], we believe that UAS-lidar should be used as a sampling tool to bridge the spatial gap between ground techniques and wall-to-wall satellite data. This may be accomplished by utilizing a two-phase sampling strategy in which regions to be covered by UAS-lidar data are sampled using ground techniques and areas to be covered by wall-to-wall satellite imagery are sampled using UAS-lidar [41]. As such, the relationship between UAS-lidar metrics and field estimated FSA at the local sample site is of prime importance for modelling generic wall-to-wall relationships.

## Conclusion

In this work, we extracted and selected suitable UAS-lidar metrics for estimating FSA at two wet Miombo woodlands sites. Four FSA (AGB, BA, DBH, and Vol) were estimated using MLR (adj-R2 >  0.79 and rRMSE <  21%). The findings show that the UAS-lidar technique reported in this study improves upon previous approaches for estimating aboveground biomass (AGB) in Miombo woodlands. In addition, the accuracy of FSA estimating models built with UAS-lidar data from a single site was compared to models generated using combined data from two separate sites. The results demonstrated that site-specific models outperformed models employing combined data. This phenomenon may be anticipated since the homogeneity of forest structure and composition is expected to be greater in a specific site than over a larger geographical region. Nevertheless, a common data model has more generality and is better suited for use over a larger geographic region.

The findings of this research give an alternative remote sensing-based FSA estimation with the accuracy needed for sustainable forest management at the local level as well as international reporting requirements such as REDD + and MVR.

However, although our research confirmed the efficacy of UAS-lidar in estimating FSA, we only considered two mature wet Miombo woodlands in similar forest environments. Therefore, it is apparent that further work on this topic is required if the full potential of UAS-lidar as a source of forest inventory data in the Miombo woodlands is to be realized. Future works should focus on estimation of FSA in the dry Miombo and hill Miombo, as well as different age groups of the regenerating Miombo woodlands, so as to have full understanding of the performance and limitations of UAS-lidar in estimating FSA across the entire spectrum of the Miombo woodlands. Furthermore, this study used area-based methods to estimate FSA from UAS-lidar. With continuous improvement of UAS-lidar sensors, increased point cloud densities per square meter, and advancement of processing technologies coupled with the open nature of the Miombo woodlands canopy, we expect future studies to focus on individual tree-based methods for estimating FSA, resulting in richer forest inventory data that would benefit sustainable management of the Miombo woodlands.

## Supporting information

S1 File UAS-lidar DTMS and sample data for Mwekera and Miengwe study sites. (ZIP)
